# Palm Tocotrienol Supplementation Enhanced Bone Formation in Oestrogen-Deficient Rats

**DOI:** 10.1155/2012/532862

**Published:** 2012-10-22

**Authors:** Ima Nirwana Soelaiman, Wang Ming, Roshayati Abu Bakar, Nursyahrina Atiqah Hashnan, Hanif Mohd Ali, Norazlina Mohamed, Norliza Muhammad, Ahmad Nazrun Shuid

**Affiliations:** Department of Pharmacology, Faculty of Medicine, Universiti Kebangsaan Malaysia, Jalan Raja Muda Abdul Aziz, 50300 Kuala Lumpur, Malaysia

## Abstract

Postmenopausal osteoporosis is the commonest cause of osteoporosis. It is associated with increased free radical activity induced by the oestrogen-deficient state. Therefore, supplementation with palm-oil-derived tocotrienols, a potent antioxidant, should be able to prevent this bone loss. Our earlier studies have shown that tocotrienol was able to prevent and even reverse osteoporosis due to various factors, including oestrogen deficiency. In this study we compared the effects of supplementation with palm tocotrienol mixture or calcium on bone biomarkers and bone formation rate in ovariectomised (oestrogen-deficient) female rats. Our results showed that palm tocotrienols significantly increased bone formation in oestrogen-deficient rats, seen by increased double-labeled surface (dLS/Bs), reduced single-labeled surface (sLS/BS), increased mineralizing surface (MS/BS), increased mineral apposition rate (MAR), and an overall increase in bone formation rate (BFR/BS). These effects were not seen in the group supplemented with calcium. However, no significant changes were seen in the serum levels of the bone biomarkers, osteocalcin, and cross-linked C-telopeptide of type I collagen, CTX. In conclusion, palm tocotrienol is more effective than calcium in preventing oestrogen-deficient bone loss. Further studies are needed to determine the potential of tocotrienol as an antiosteoporotic agent.

## 1. Introduction

Osteoporosis is an important age-related disease constituting a major health problem especially among postmenopausal women. In women, the rate of bone loss increases dramatically after menopause or ovariectomy due to oestrogen deficiency. Thus, there is an increased rate of activation of remodeling sites resulting in the decrease of both trabecular and cortical bones. In addition, oestrogen deficiency has been associated with oxidative stress because oestrogen is an antioxidant with radical-scavenging properties. The levels of lipid peroxidation (LPO) and hydrogen peroxide (H_2_O_2_) were increased and enzymatic antioxidants like superoxide dismutase (SOD), glutathione peroxidase (GPx), glutathione S transferase (GST) were decreased in the femur of ovariectomized rats [1]. Oxidative stress has been shown to stimulate osteoclast differentiation and function as well as inhibit osteoblast differentiation. Glutathione peroxidase 1 (Gpx1) is the main antioxidant enzyme expressed in osteoclasts and is responsible for the degradation of hydrogen peroxide. Its overexpression in the osteoclastic cell line RAW264.7 prevents RANKL-induced osteoclastogenesis [2]. Hydrogen peroxide is essential for oestrogen deficiency bone loss and osteoclast formation, suggesting a crucial role for hydrogen peroxide in osteoclast formation. 17*β*-estradiol stimulates Gpx1 expression in bone-marrow-derived osteoclasts, and oestrogen deficiency was a key step in the reactive oxygen species (ROS-) mediated stimulation of TNF*α* expression, leading to enhanced osteoclastogenesis and bone resorption [3]. Plasma total oxidative status (TOS) and oxidative stress index (OSI) value were significantly higher, and plasma total antioxidant status (TAS) level was lower in patients than in healthy controls (*P* < 0.001 for all). There was a significant negative correlation between OSI and bone mineral density (BMD) in lumbar and femoral neck region (*r *= 0.63, *P* < 0.001; *r* = 0.40, *P* = 0.018). These findings indicated that increased osteoclastic activity and decreased osteoblastic activity may be associated with an imbalance between oxidant and antioxidant status in postmenopausal osteoporosis [4]. Our own earlier studies showed that ferric nitrilotriacetate (Fe-NTA), a strong oxidising agent, increased osteoclast number and reduced osteoblast number in rat femoral bone. Trabecular thickness was also lower in the group treated with Fe-NTA, compared to the normal control [5]. In light of these findings, it is hypothesized that antioxidants can prevent postmenopausal bone loss.

Palm oil vitamin E consists of tocopherols and tocotrienols. Each of them has four stereoisomers respectively, namely, *α*-, *β*-, *γ*-, and *δ*-tocopherols (T) and tocotrienols (T3). Tocopherols contain a saturated phytol side chain in the chroman ring and a saturated hydrocarbon chain. On the other hand, tocotrienols contain three double bonds in the side chain and an unsaturated hydrocarbon chain. Palm tocotrienol at the dose of 100 mg/kg body weight significantly reduced the thiobarbituric acid reactive substances (TBARSs) level in the femur with a significant increase in glutathione peroxidase activity compared to the age-matched control group. These were not observed in the *α*-tocopherol group. This indicated that tocotrienol has better antioxidant activity in bone than *α*-tocopherol [6]. Our earlier studies have shown that tocotrienol was able to prevent and even reverse osteoporosis in oestrogen deficiency, testosterone deficiency, glucocorticoid excess, and nicotine exposure [7–13]. The dose of tocotrienol, 60 mg/kg, had been established as the optimum dose for bone protection which we have established in our previous studies [5, 13–15].

Calcium is the most abundant mineral in the body. It is highly consumed through diet. The richest source of calcium includes cheeses and low-fat dairy products such as milk, yogurt, and tau-fu. It is essential for the development and maintenance of strong bones and teeth. Calcium supplementation has been used as treatment for osteoporosis. In adults with a baseline calcium intake of 500–900 mg/day, increasing or supplementing this intake by a further 500–1000 mg/day has a beneficial effect on bone mineral density [16]. 

Quantitative changes in bone turnover can be assessed easily and noninvasively by the measurement of serum and urinary biochemical markers; the most sensitive markers include serum osteocalcin, bone specific alkaline phosphatase, the N-terminal propeptide of type I collagen for bone formation, and the crosslinked C-(CTX) and N-(NTX) telopeptides of type I collagen for bone resorption [17]. In this study we assayed serum osteocalcin and CTx. The serum cross-linked C-telopeptide of type I collagen (CTX) is a marker of osteoclast activity and is used to assess the level of bone resorption. These peptide fragments are detected by the radioimmunoassay technique. Serum osteocalcin is a major noncollagenous protein that is synthesized by osteoblasts. It plays an important role in the regulation of bone growth and in the correct deposition of the minerals in the matrix. Its expression follows the proliferative phase of osteoblastic differentiation, so it can be considered a marker of mature osteoblasts. Bone histomorphometry is the quantitative study of the microscopic organization and structure of bone tissue by computer-assisted analysis of images formed by a microscope. Bone histomorphometry contains three sets of parameters, namely, structural, static, and dynamic parameters. In this study we studied the dynamic bone histomorphometry which measures the rate of bone growth over time [18]. 

The aim of this study was to compare between the effects of palm tocotrienols and calcium supplementation on bone metabolism ovariectomised (oestrogen-deficient) rats. The results of this study will indicate whether the tocotrienols are better than calcium as supplements to prevent osteoporosis in the postmenopausal state.

## 2. Materials and Methods

### 2.1. Animals and Treatment

Thirty-two female Sprague-Dawley rats (four months old) weighing between 180 and 200 g were obtained from the Laboratory Animals Resource Unit, Universiti Kebangsaan Malaysia. The rats were randomly assigned into groups of sham-operated (SHAM), ovariectomised-control (OVC), ovariectomised and given 60 mg kg^−1^ of tocotrienol mixture (OV+T), and ovariectomised and given 1% calcium in drinking water *ad libitum* (OV+Ca). Each group has eight rats. Two to three rats were kept in a cage under 12-hour natural light/dark cycles. All rats received normal rat chow from Gold Coin (Port Klang, Selangor, Malaysia). Calcium content of the rat chow as given by the supplier was between 0.8 1.2% [14].

All the groups except the Ovx+Ca group were given deionised water *ad libitum*. The palm tocotrienol mixture was a gift from Carotech Bhd. (Ipoh, Malaysia) consisting of *α*-tocotrienol 24.67%, *γ*-tocotrienol 38.955%, *δ*-tocotrienol 4.55%, and *α*-tocopherol 20.11%. It was diluted in olive oil (Bertolli, Italy) and given via oral gavage at the dose of 60 mg kg^−1^ body weight daily at 9 am for 8 weeks. Calcium supplementation was administered as 1% calcium in drinking water ad libitum [19]. For measurement of dynamic histomorphometric parameters, the bone samples were double labelled by intraperitoneal injections of a fluorochrome, calcein, at 9 days and 2 days before sacrifice [18]. The fluorochrome was incorporated into the active formation sites in the bone and can be visualised as fluorescent lines by fluorescent microscopy.

A baseline control group (BC) of eight rats was given the calcein injections upon receipt and sacrificed without any treatment. The right femora were dissected and prepared for dynamic histomorphometric studies.

### 2.2. Blood and Bone Sampling

Blood samples were collected before the start and after eight weeks of treatment from the retroorbital vein after anesthetizing the rats with ether. After 3 hours in room temperature, the blood was centrifuged at 3000 rpm for 10 min and the serum was stored at −70°C. The left femora were dissected out and fixed with 70% alcohol.

### 2.3. Bone Biochemical Markers

Bone biochemical markers of serum osteocalcin and C-terminal telopeptide of type 1 collagen (CTX) were measured before and after the treatment using an ELISA reader (VERSAmax, Sunnyvale, USA). The kits used were rat osteocalcin ELISA (Biomedical Technologies, Herlev, Denmark) and Ratlaps ELISA CTX-1 (Nordic Biosciences, IDS, UK).

### 2.4. Bone Histomorphometry

The dynamic bone histomorphometric parameters were measured according to The American Society of Bone Mineral Research Histomorphometry Nomenclature Committee 1987 [18]. The left femora were dissected out and fixed with 70% ethanol. After one week, the femora were cut sagittally at the epiphyseal and metaphyseal area. The femora were then embedded in methyl methacrylate (Osteo-Bed Bone Embedding Kit; Polysciences, USA) and sectioned at 9 *μ*m thickness using a microtome (Leica RM2155, Wetzlar, Germany). The unstained bones were analyzed using an image analyzer Pro-Plus (Media Cybernetics, Silver Spring, MD, USA) and a fluorescence microscope (Nikon Eclipse 80 *μ*, Japan). The dynamic bone histomorphometric parameters include single-labeled surface/bone surface (sLS/BS), double-labeled surface/bone surface (dLS/BS), mineralizing surface/bone surface (MS/BS), bone formation rate/bone surface (BFR/BS), and Mineral Apposition Rate (MAR). The primary parameters are the single-labeled surface (sLS) and double-labeled surface (dLS). The dLS represented bone growth in seven days, while the sLS was actually two calcein labels superimposed on each other due to insignificant bone growth. The sLS/BS and dLS/BS parameters are obtained using the Weibel grid as described by Freere and Weibel [20]. The other parameters were derived as follows: MS/BS = [dLS + 1/2 sLS]/BS and BFR/BS = [dLS + 1/2 sLS X MAR]/BS. The MAR was calculated by obtaining the mean interlabel distance using the Pro Plus image anaylzer and dividing it by seven days, which is the duration of time between the calcein injections. The measurements were performed at the metaphyseal region which is rich in trabecular bone. This secondary spongiosa area is located 3 to 7 mm from the lowest point of the growth plate and 1 mm from the lateral cortex, excluding the endocortical region [21].

### 2.5. Statistical Analyses

The Kolmogorov-Smirnoff tests were used for normality testing. Following that the Kruskal-Wallis andMann-Whitney *U* tests were used for comparison between treatment groups since the data were not normally distributed. To compare data between before and after treatment, the Wilcoxon Rank Sign test was used. Data analysis was carried out using the Statistical Package for Social Sciences (SPSSs) version 19.0 software. The results were expressed as mean values ± standard error of the mean (SEM).

This study was approved by Universiti Kebangsaan Malaysia Animal Ethics Committee (UKMAEC) certificate no: FP/FAR/2010/NAZRUN.

## 3. Results

### 3.1. Dynamic Bone Histomorphometry

**Figure 1:**
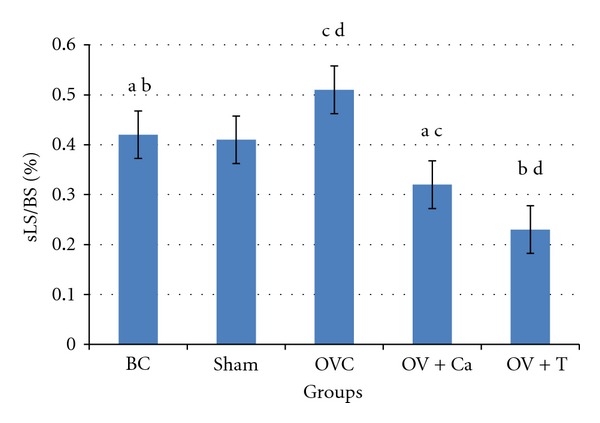
Single-labeled surface (sLS/BS). BC: baseline control group. Sham: sham-operated rats given deionised water and vehicle olive oil. OVC: ovariectomised rats given deionised water and vehicle olive oil. Ov+Ca: ovariectomised rats given 1% calcium in deionised water and vehicle olive oil. Ov+T: ovariectomised rats given deionised water and palm tocotrienol. Same alphabet indicates significant difference between the groups (*P* < 0.05).

sLS indicates that there was poor bone growth, such that the two calcein labels were superimposed on each other.  Figure 1 showed that increased percentage of sLS in the OVC group, while both calcium and tocotrienol significantly reduced the percentage of sLS.

**Figure 2:**
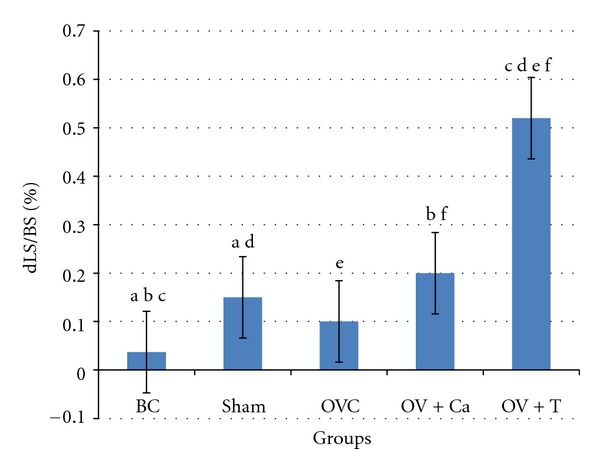
Double-labeled surface (dLS/BS). BC: baseline control group. Sham: sham-operated rats given deionised water and vehicle olive oil. OVC: ovariectomised rats given deionised water and vehicle olive oil. Ov+Ca: ovariectomised rats given 1% calcium in deionised water and vehicle olive oil. Ov+T: ovariectomised rats given deionised water and palm tocotrienol. Same alphabet indicates significant difference between the groups (*P* < 0.05).

dLS indicates bone growth. The interlabel distance indicates bone growth in seven days. Ovariectomy did not significantly change the percentage of dLS compared to BC and Sham. However, tocotrienol significantly increased percentage of dLS more than all the other groups (Figure 2).

**Figure 3:**
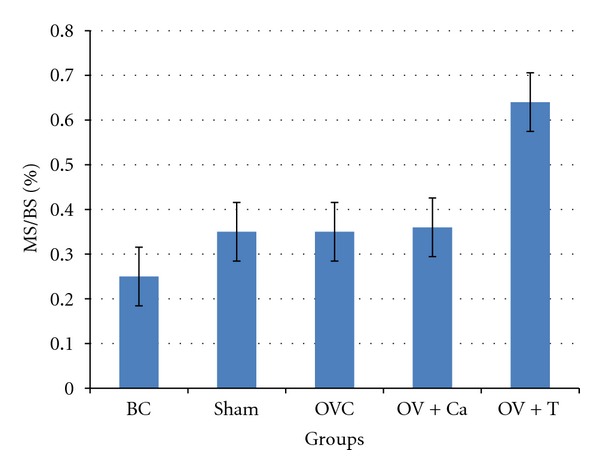
Mineralising surface (MS/BS). BC: baseline control group. Sham: sham-operated rats given deionised water and vehicle olive oil. OVC: ovariectomised rats given deionised water and vehicle olive oil. Ov+Ca: ovariectomised rats given 1% calcium in deionised water and vehicle olive oil. Ov+T: ovariectomised rats given deionised water and palm tocotrienol. No significant difference was seen between the groups at *P* < 0.05.

No significant differences were seen between groups in the percentage of MS/BS. (Figure 3).

**Figure 4:**
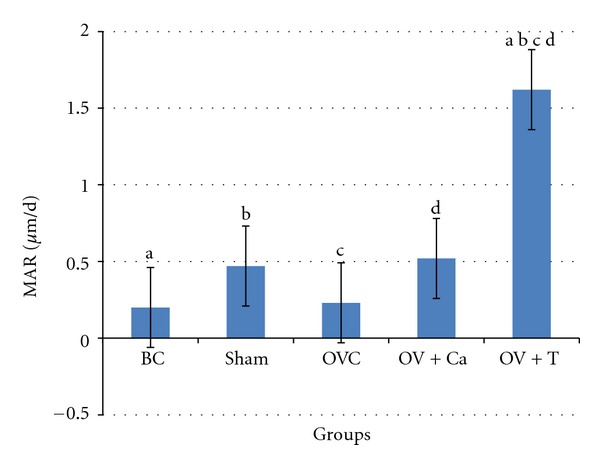
Mineral apposition rate (MAR). BC: baseline control group. Sham: sham-operated rats given deionised water and vehicle olive oil. OVC: ovariectomised rats given deionised water and vehicle olive oil. Ov+Ca: ovariectomised rats given 1% calcium in deionised water and vehicle olive oil. Ov+T: ovariectomised rats given deionised water and palm tocotrienol. Same alphabet indicates significant difference between the groups (*P* < 0.05).

The MAR was higher in the Ov+T group compared to all the other groups. Calcium supplementation to the ovariectomised rats (Ov+Ca) did not increase the MAR (Figure 4).

**Figure 5:**
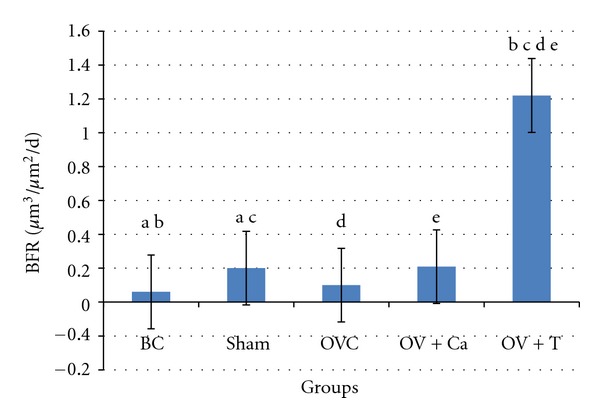
Bone formation rate (BFR/BS). BC: baseline control group. Sham: sham-operated rats given deionised water and vehicle olive oil. OVC: ovariectomised rats given deionised water and vehicle olive oil. Ov+Ca: ovariectomised rats given 1% calcium in deionised water and vehicle olive oil. Ov+T: ovariectomised rats given deionised water and palm tocotrienol. Same alphabet indicates significant difference between the groups (*P* < 0.05).

BFR was higher in the Ov+T group compared to all the other groups. The Ov+Ca group did not differ from the OVC group (Figure 5).

**Figure 6:**
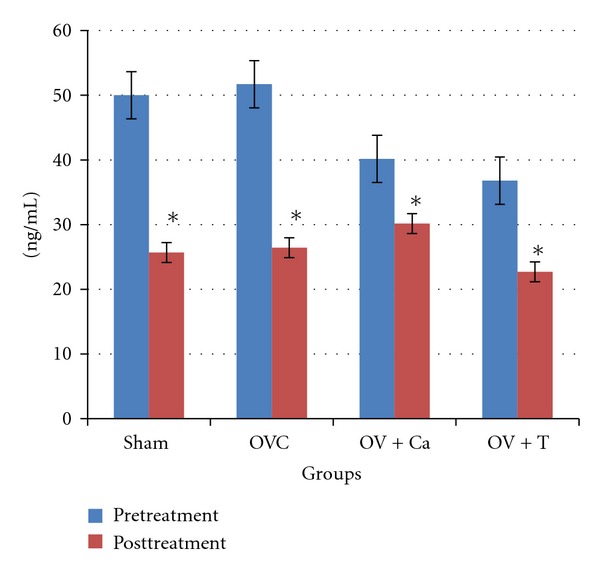
Serum C-terminal telopeptide of type 1 collagen (CTX). Sham: sham-operated rats given deionised water and vehicle olive oil. OVC: ovariectomised rats given deionised water and vehicle olive oil. Ov+Ca: ovariectomised rats given 1% calcium in deionised water and vehicle olive oil. Ov+T: ovariectomised rats given deionised water and palm tocotrienol. *indicates significant difference before and after treatment (*P* < 0.05).

The CTX levels were significantly lower in all the groups after treatment. However, there were no significant differences before and after treatment for all the groups (Figure 6).

**Figure 7:**
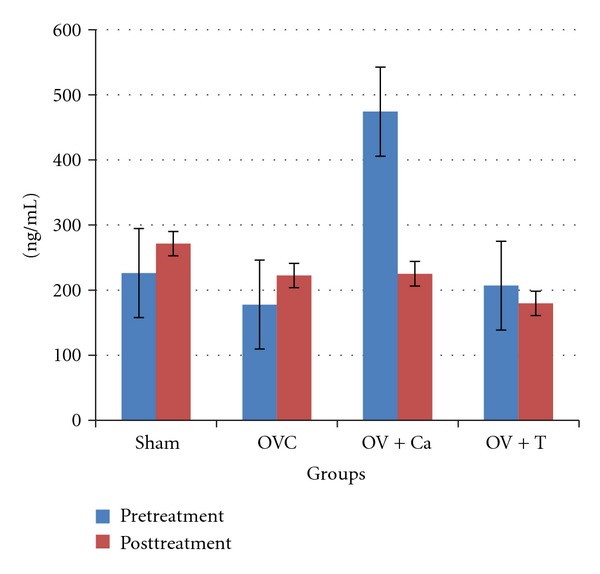
Serum osteocalcin. Sham: sham-operated rats given deionised water and vehicle olive oil. OVC: ovariectomised rats given deionised water and vehicle olive oil. Ov+Ca: ovariectomised rats given 1% calcium in deionised water and vehicle olive oil. Ov+T: ovariectomised rats given deionised water and palm tocotrienol. No significant difference was seen between the groups at *P* < 0.05.

There were no significant differences in serum osteocalcin levels seen between groups as well as within groups (Figure 7).

## 4. Discussion

During early adulthood, bone mass is stable until menopause. For about the first five years after menopause, women lose bone mass at the rate of about 2% to 3% per year and then continue to lose about 1% of bone mass per year to the end of life. Hence, postmenopausal women are prone to osteoporosis [16]. 

Early reports by others showed that calcium supplementation in postmenopausal women for two months was shown to be effective in preventing osteoporosis. Markers of bone turnover were reduced [22]. In the present study, calcium supplementation was given to ovariectomised rats. However, no improvement in the dynamic histomorphometry was observed. The position statement on calcium and bone health by the Australian and New Zealand Bone and Mineral Society, Osteoporosis Australia, and the Endocrine Society of Australia states that the effect of calcium supplementation on bone health is modest, as shown by increases in BMD and reductions in excessive bone turnover. However, the relative risk reduction for osteoporotic fracture is likely to be no more than 10%–20%. Although inadequate calcium intake is likely to be deleterious to bone, calcium intake significantly *above *the recommended level is unlikely to achieve additional benefit for bone health [16]. This is in agreement with our study which did not find any significant improvement in bone formation in rats supplemented with calcium.

Palm vitamin E contains a mixture of tocopherols and tocotrienols but with a higher percentage of tocotrienol. Our preparation consisted of 80% tocotrienol and only 20% tocopherol. Our previous animal studies found that both tocotrienols and tocopherols were able to prevent the decline in bone density due to various factors. However, the tocotrienols were found to have better efficacy in some studies [10, 13, 23]. In fact, tocotrienol was also shown to improve bone biomechanical strength better than *α*-tocopherol [15]. This may be because tocotrienol demonstrated better antioxidant properties in bones compared to *α*-tocopherol [6]. A study by Fujita et al. [24] found that *α*-tocopherol actually increases bone resorption by stimulating osteoclast fusion, an effect that was not shown by *α*-tocotrienol. This effect of *α*-tocopherol was thought not to be due to its antioxidant properties, since other antioxidants, including *α*-tocotrienol, simultaneously tested, did not show that effect. In this study, our preparation was mainly tocotrienol, and it showed significantly better effects on bone formation in oestrogen-deficient rats compared to calcium. Therefore, we postulate that the beneficial effects were due to the tocotrienol component and not *α*-tocopherol, since our previous studies showed that *α*-tocopherol was less effective. Furthermore, the study of Fujita et al. [24] indicated that *α*-tocopherol was harmful to bone. Thus, it is important to remove the *α*-tocopherol component from palm vitamin E, leaving only the beneficial tocotrienols in place.

In this study, the dynamic histomorphometry clearly showed that tocotrienols increased bone formation and reduced bone resorption. However, no significant changes in the serum bone biomarkers were seen even though CTX was significantly lower in all the groups after treatment compared to before treatment was started. This may be because serum biomarkers are subject to circadian rhythm, therefore variation in serum levels may contribute to the insignificant results seen. Another reason may be that bone remodeling is a dynamic process, thus serum biomarkers are in a constant state of fluctuation. Furthermore, the technique may not be sensitive enough to detect small changes in nanograms per milliliter. The antiosteoporotic effects of tocotrienol are better illustrated in the dynamic histomorphometric parameters since findings in bone are more stable and lasting compared to serum findings. Similar biomarker findings were seen in our previous study [25].

There were several limitations in the current study which should be noted. The rats in group OV+Ca were treated with 1% calcium in drinking water, *ad libitum* daily for two months. One gram of calcium was powdered and mixed with 99 mL of deionised water which the rats drank when needed. In this way, the amount of calcium consumption could not be quantified. However, this method was selected since it did not cause any trauma to the animals. This is unavoidable since the tocotrienol and vehicle were administered by daily oral gavage. To administer calcium in this way will mean two oral gavages in a day which might traumatize the animal too much. We have successfully used this mode of administration of calcium in our earlier studies [14, 26, 27]. In addition, Sanders et al. [16] mentioned that the benefits of calcium treatment are more consistent in late postmenopausal women than in perimenopausal women, perhaps because of greater variation in the rate of bone loss amongst perimenopausal women. In the present study, the menopausal state in rats was induced by ovariectomy. After two weeks of ovariectomy, treatment with calcium was started. Therefore these ovariectomised rats may be considered to be in the early postmenopausal state. This may contribute to the results seen in our study, which showed that calcium supplementation did not significantly affect bone resorption and formation. 

In conclusion, tocotrienols increased bone formation rate in oestrogen-deficient ovariectomised rats, while calcium did not. Further studies are needed to determine the potential of tocotrienol as an antiosteoporotic agent.
